# The Vascular‐Immune Cells Crosstalk and Microenvironment in Pulmonary Hypertension

**DOI:** 10.1002/cph4.70110

**Published:** 2026-02-13

**Authors:** Lola Navarro‐Llinares, Bertha García‐León, Laura de la Bastida‐Casero, Eduardo Oliver

**Affiliations:** ^1^ Center for Biological Research Margarita Salas (CIB), CSIC Madrid Spain; ^2^ Spanish National Center for Cardiovascular Research (CNIC) Madrid Spain; ^3^ Centro de Investigación Biomédica en Red de Enfermedades Cardiovasculares (CIBERCV) Madrid Spain

**Keywords:** crosstalk, endothelial cells, immune cells, microenvironment, pulmonary hypertension, smooth muscle cells, vascular remodeling

## Abstract

Pulmonary hypertension is a disease of the lung vasculature, which eventually leads to heart dysfunction. Despite the advances in the discovery and development of new treatments, most of them are aimed to palliate symptoms. Thus, it is crucial to identify novel potential targets directly acting on vascular remodeling. Further understanding of this pathological event is needed to completely describe the disease's mechanisms and effectively generate therapeutical strategies able to cure the disease. Here, we propose an integrative perspective of studying pulmonary hypertension based on the interactions between the vascular cells and their surroundings. This review describes the crosstalk between vascular cells and immune cells in the diseased vasculature, highlighting the central role of this axis and the importance of healthy cell‐to‐cell communication. Furthermore, we discuss the possibility of considering the pulmonary microenvironment as a key pathological factor in pulmonary hypertension.

## Graphical abstract

This review synthesizes current evidence showing how vascular–immune cell crosstalk and microenvironmental cues shape pulmonary vascular remodeling in pulmonary hypertension. We provide an integrative perspective that positions disrupted cellular communication as a central driver of disease progression and a promising source of future therapeutic targets.

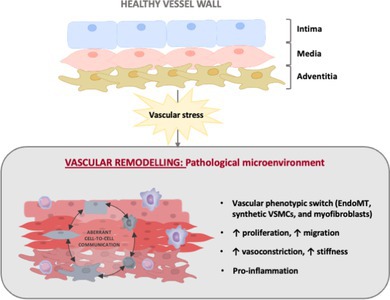

## 1 Introduction

Pulmonary hypertension (PH) is a group of disorders characterized by a pathological chronic increase in the mean pulmonary arterial pressure (mPAP > 20 mmHg at rest) (Johnson et al. 2023). According to the World Symposium on Pulmonary Hypertension (WSPH), PH is classified in five clinical subgroups based on its etiology: group 1, pulmonary arterial hypertension (PAH); group 2, PH due to left heart disease; group 3, PH associated with lung disease; group 4, PH due to pulmonary artery obstructions; and group 5, PH with unclear or multifactorial mechanisms (Humbert et al. 2022). Despite the underlying trigger, they all manifest abnormal vasoconstriction, cell proliferation, and inflammation in the lung vasculature, driving what is known as vascular remodeling. This aberrant remodeling of pulmonary small arterioles contributes to the elevated mPAP, which leads to progressive right heart hypertrophy and, eventually, heart failure and premature death (Gallardo‐Vara et al. 2023; Zhang and Wang 2023; Mandras et al. 2020).

Widely used treatments are mainly vasodilators with the ultimate goal of mitigating right ventricle overload. However, they only partially palliate symptoms and do not effectively cure the disease (Guignabert et al. 2015; de Martin Miguel et al. 2023; Kondo et al. 2019). Even though a lot of progress has been made related to alleviating PH symptoms among patients, there has been little improvement in mortality rates (Nabeh et al. 2024). Evidence highlights the relevance of vascular remodeling in PH pathogenesis; still, only one recent treatment, sotatercept, has focused on this pathological hallmark as a potential cure of the disease (Gallardo‐Vara et al. 2023; Guignabert et al. 2015; Kumar et al. 2024; Humbert et al. 2021; Condon et al. 2022). In this context, understanding and describing the vascular remodeling process is crucial to identify novel targets and develop novel therapeutical interventions.

Recent research highlights the importance of cell‐to‐cell communication between vascular and immune cells in the vascular remodeling process, which potentially prompts the generation of a pathological microenvironment (Guignabert et al. 2015; Méndez‐Barbero et al. 2021; Humbert et al. 2019). Our goal with this article is to review the current knowledge on the complex vascular pathobiology of PH with special focus on cellular interactions among endothelial cells (ECs), smooth muscle cells (SMCs), fibroblasts, and inflammatory cells. Further understanding of these mechanisms offers a novel integrative perspective of the disease that should help in developing therapeutic strategies to counteract this devastating disease.

## 2 Basis on the Vascular Remodeling Process in PH

**FIGURE 1:**
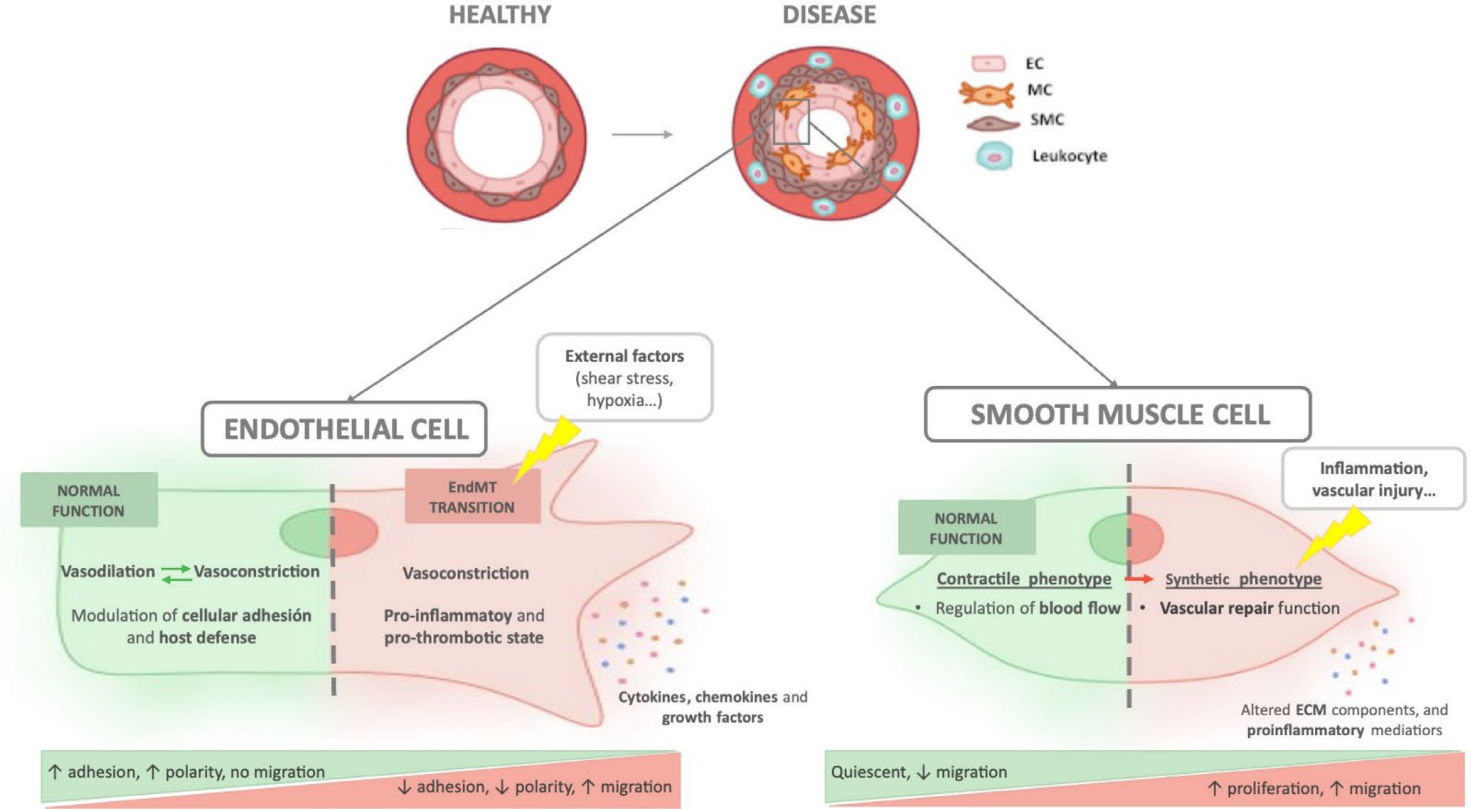
Endothelial and smooth muscle cells diseased phenotypes. Illustration shows comparisons between the healthy function of endothelial and smooth muscle cells versus their pathological status. As observed, endothelial cells, under physiological conditions, maintain a balanced vasoactive modulation and regulate cellular adhesion and host defense. However, under the exposure of certain external stimuli, they undergo a transition towards a mesenchymal phenotype, exhibiting higher production of vasoconstrictors and a pro‐inflammatory and pro‐thrombotic state. These events characterize the dysfunctional endothelium, which secretes molecules such as chemokines and cytokines that exacerbate the vascular remodeling. On the other hand, smooth muscle cells suffer from a shift towards a synthetic phenotype, responsible for a vascular repair function, increasing their proliferative and migratory capacities.

PH is a complex disease that affects the pulmonary circulation. The main histological hallmark of this disease implies the abnormal growth and thickening of the three concentric vessel layers (intima, media, and adventitia, from the lumen to the outside) with subsequent partial or complete obstruction of the lumen. Its pathological features include endothelial dysfunction, aberrant vascular wall proliferation, and inflammation (Figure 1) (Zhang and Wang 2023).

### 2.1 Endothelial Dysfunction

ECs constitute the innermost layer of blood vessels and modulate the vascular tone, fluid homeostasis, cellular adhesion, and host defense. They are constantly exposed to different stimuli and external factors such as mechanical injury, shear stress, viral infections, or chemical agents that can trigger endothelial activation. Under this situation, ECs release cytokines, chemokines, and growth factors that, if sustained in time, lead to endothelial dysfunction (Méndez‐Barbero et al. 2021). This dysfunction is characterized by a shift to a vasoconstrictive, pro‐inflammatory, and pro‐thrombotic state.

It has been hypothesized that vascular remodeling in pulmonary vascular disease begins with the early apoptosis of ECs, which leads to the proliferation of apoptosis‐resistant ECs (Marshall et al. 2018). Additionally, these ECs undergo a trans‐differentiation process, known as endothelial to mesenchymal transition (EndMT), consisting of a progressive loss of endothelial traits and an evolution into a mesenchymal phenotype. This transition involves changes in morphology, loss of cell–cell junctions and polarity, and acquisition of migratory, proliferative, and contractile cellular properties (Hall et al. 2024; Piera‐Velazquez and Jimenez 2019). These changes underlie the pathogenesis of different diseases such as hypertension, atherosclerosis, or chronic heart failure, among others (Méndez‐Barbero et al. 2021; de la Bastida‐Casero et al. 2024).

### 2.2 Vascular Smooth Muscle Cells Hyperplasia and Hypertrophy

On the other hand, the medial layer is mainly composed of vascular smooth muscle cells (VSMCs). These cells are heterogeneous in the normal pulmonary circulation, as they show high phenotypic plasticity, and can shift from a contractile state to a synthetic state, regulating their proliferative, migratory, and inflammatory capacities (Méndez‐Barbero et al. 2021; Stenmark et al. 2018).

Under physiological conditions, VSMCs exhibit predominantly a contractile phenotype and are quiescent and differentiated cells that regulate the blood flow and vessel diameter. However, they are not stable cells and can turn into a high proliferative/synthetic phenotype in response to different stimuli, such as inflammation or vascular injury (Bkaily et al. 2021). Hence, the proliferative VSMCs are responsible for vascular repair and have high synthetic, growth, and migratory activities (Bkaily et al. 2021; Nogueira‐Ferreira et al. 2014).

During the vascular remodeling process in PH, there is a redistribution of VSMCs with an enriched presence of the synthetic phenotype, which exhibits an altered production of extracellular matrix (ECM) components, proinflammatory molecules, and growth factors (Crnkovic et al. 2022). The VSMCs migrate to generally non‐muscularized distal pulmonary arterioles and undergo hypertrophy in proximal arteries (Gallardo‐Vara et al. 2023). Thus, the thickening of the smooth muscle layer is marked by an increased number and hypertrophy of VSMCs, which exhibit enhanced chemokine and cytokine production and alterations in ECM protein production and degradation (Stenmark et al. 2018).

### 2.3 Changes in the Adventitia Layer and Inflammation

The adventitia layer is the most heterogeneous layer of the vessel wall, composed of fibroblasts, immune cells, and progenitor cells, and also suffers a substantial growth in PH (Gallardo‐Vara et al. 2023). This thickening is due to an increase in collagen and ECM deposition, proliferation of resident fibroblasts, and recruitment of circulating immune cells, among others.

Fibroblasts, the main cellular component, are widely known for their role maintaining tissue structure through the deposition and remodeling of ECMs and organizing functional tissue networks (Kumar et al. 2021). They can be activated in response to different stimuli, including hypoxia, mechanical stress, and inflammation, and they differentiate into myofibroblasts, characterized by the expression of smooth muscle cell markers (Zhang, Li, et al. 2024). This transition, although it is a component of the normal wound‐healing process, can lead to persistent fibrosis due to excessive proliferation and deposition of ECM. Specifically, fibroblasts from the adventitia layer of pulmonary arteries exhibit the earliest proliferative, apoptosis‐resistant, fibrotic, and inflammatory responses to vascular stress, highlighting their role in PH development (Kumar et al. 2021; Zhang, Li, et al. 2024).

Importantly, the fibroblasts from remodeled pulmonary arteries exhibit a proinflammatory reprogramming. They are able to release chemokines and cytokines that recruit immune cells and modulate their action (Liu et al. 2022). All of these, together with the overproduction of chemokines and cytokines from VSMCs and ECs, establish a microenvironment prompt to inflammation. In this way, persistent inflammation is common in PH, and it has gradually gained attention as an important component of PH disease (Crnkovic et al. 2025; Plecitá‐Hlavatá et al. 2023).

Both innate and adaptive immune cells contribute to this inflammatory response. Example of this is found in PAH patients and corresponding animal models, which show an accumulation and infiltration of immune cells, including innate and adaptive cells in their lungs, as well as increased inflammatory mediators in the circulation in early stages of the disease (Liu et al. 2022; Luo and Qiu 2022). In this way, some features include mast cells' accumulation in diseased lungs, macrophages and monocytes' infiltration in vascular lesions and remodeled vessels, and dendritic cells' presence in adventitial and vascular lesions (Kuebler et al. 2018). Adaptive immune responses are also implicated in PH pathogenesis, for instance, with B‐cells' accumulations in perivascular spaces, plexiform lesions, and tertiary lymphoid tissues near remodeled vessels. This infiltration is associated with elevated inflammatory cytokines (interleukins (IL) including IL‐1β, IL‐4, IL‐6, etc.; and tumor necrosis factor TNFα) in both pulmonary and systemic circulation (Liu et al. 2022; Luo and Qiu 2022).

## 3 Cellular Crosstalk in PH

Cellular crosstalk plays a key role in the development and progression of PH, where the dynamic interaction between different cell types in the pulmonary vasculature promotes the pathological vascular remodeling. In this context, the aberrant pathological phenotypes of ECs and VSMCs are critical.

On the one hand, endothelium dysfunction is characterized by endothelial injury, excessive EC proliferation, and EndMT. During EndMT, ECs progressively acquire myofibroblastic phenotypes, enabling them to invade the intima and contribute to vascular remodeling. This transformation disrupts normal vascular homeostasis and amplifies pathological signaling pathways, further stimulating vasoconstriction and inflammation (Good et al. 2015; Morrell et al. 2009). On the other hand, VSMCs, which normally regulate vascular tone and blood flow through their contractile function, become highly plastic under pathological conditions. In PH, they exhibit cancer‐like characteristics, including excessive proliferation, resistance to apoptosis, and enhanced synthetic activity. These changes lead to hypertrophy and progressive narrowing of the pulmonary arterial lumen (Stenmark et al. 2018).

**FIGURE 2:**
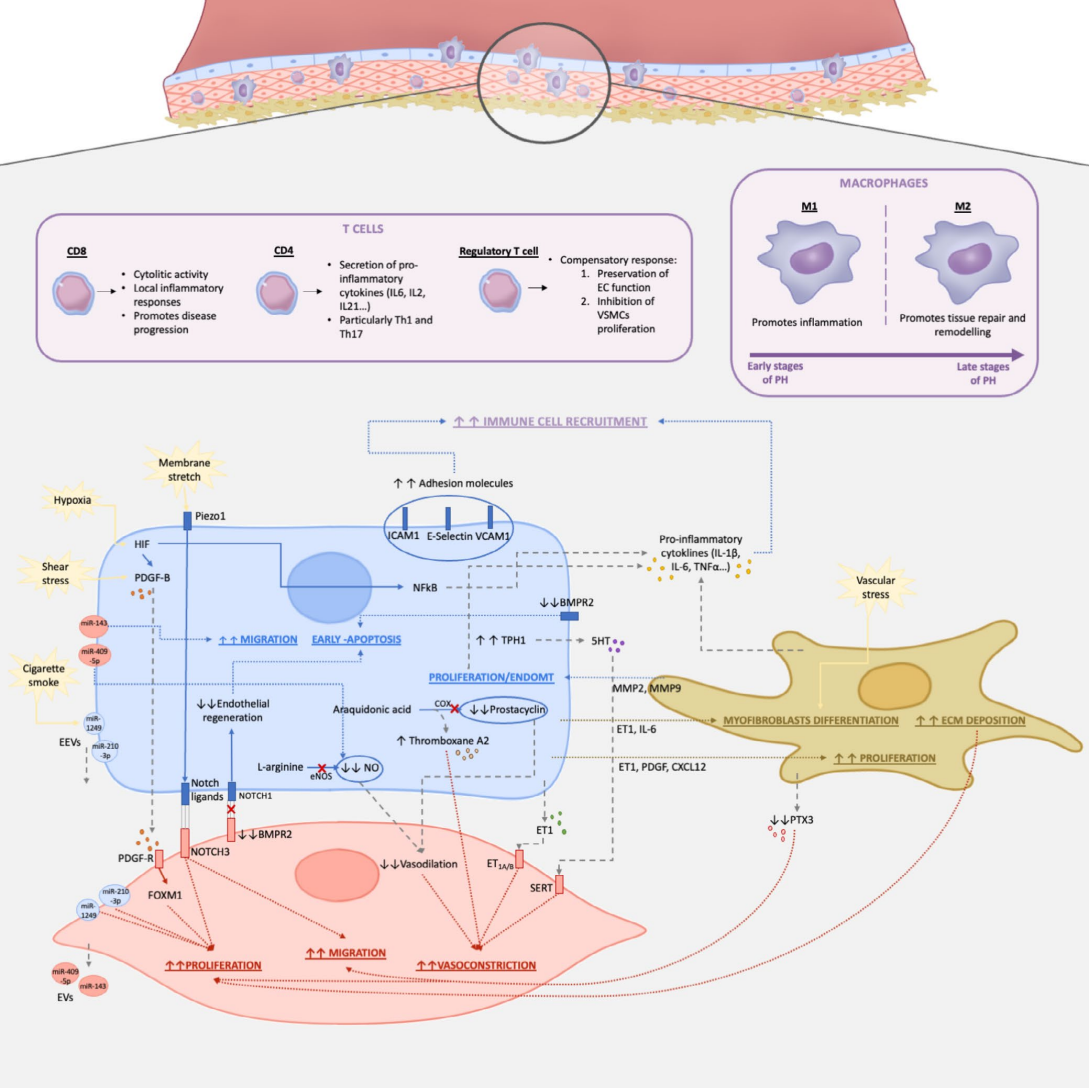
Cell‐to‐cell communication between ECs, VSMCs, fibroblasts and immune cells in vascular remodeling. Illustration shows interactions between ECs (in blue), VSMCs (in red), fibroblasts (in brown) and immune cells (in purple). Speech bubbles in yellow indicate external stimuli affecting the target cells; gray dotted lines designate molecules that are secreted; blue, red, and brown dotted lines indicate the association between a molecule or signaling pathway and its pathogenic effects in ECs, VSMCs, and fibroblasts, respectively; straight blue and red lines represent downstream effectors in ECs and VSMCs, respectively.

In this complex microenvironment, cellular crosstalk also extends to interactions with other cell types, including fibroblasts and immune cells, which collectively drive inflammation (Thompson and Lawrie 2017) (Figure 2). Understanding these interactions is needed to identify novel therapeutic targets that can disrupt the pathological signaling pathways driving PH progression.

### 3.1 ECs to VSMCs Communication

Cellular communication between ECs and VSMCs is extremely important in vascular homeostasis. This crosstalk is established in different ways, including direct cell contact, paracrine signaling through soluble factors, and extracellular vesicles. In this way, to maintain the contractile phenotype of VSMCs, ECs are continuously releasing vasoactive molecules, such as prostanoids, arachidonic acid, and nitric oxide (NO). When there is a vascular injury or loss of endothelial integrity, the normal crosstalk is disturbed, triggering the VSMCs' phenotypic and functional changes, as well as inflammation (Méndez‐Barbero et al. 2021).

#### 3.1.1 Soluble Factors Targeting VSMCs Proliferation and Vasoconstriction

Under hypoxic conditions, as in PH progression, hypoxia‐inducible factors (HIF) are overexpressed and stimulate the production of different signaling molecules. In ECs, HIFs promote the expression of many growth factors, including the platelet‐derived growth factor‐B (PDGF‐B). PDGF‐B is a strong VSMCs mitogen, as it induces forkhead box M1 (FoxM1) expression promoting their proliferation. FOXM1 is a transcriptional factor commonly overexpressed in VSMCs of small arterioles in PAH patients and in the monocrotaline‐induced PH rat model (Gallardo‐Vara et al. 2023; Sakao and Tatsumi 2011). Other studies have also demonstrated that shear stress can upregulate the PDGF‐B production in the dysfunctional endothelium (Zhang and Wang 2023; Méndez‐Barbero et al. 2021). Targeting PDGF receptors with antagonists has been shown to reverse vascular remodeling in experimental PH models, emphasizing the importance of this pathway in EC‐VSMC communication (Condon et al. 2022; Sakao and Tatsumi 2011).

Endothelial dysfunction also carries a decompensation in vasoactive modulators. Endothelin‐1 (ET1) is a peptide secreted by ECs that acts as a vasoconstrictor and induces migration and proliferation of vascular cells. Specifically, VSMCs express both membrane receptors of ET1 (ETA and ETB). Circulating ET‐1 levels are elevated in all animal models and human forms of PH (Shimoda and Laurie 2013). Another important vasoactive molecule is serotonin (5‐hydroxytryptamine, 5‐HT), which has been reported to be upregulated in the pulmonary arterial endothelium of PAH patients. Specifically, ECs show increased expression of tryptophan hydroxylase 1 (TPH1), responsible for the synthesis of 5‐HT, and 5‐HT itself. Then, serotonin enters VSMCs through the serotonin transporter (SERT) and the 5‐HT1B receptor, which activates contractile and proliferative signaling pathways (MacLean et al. 2022; Gu et al. 2017; Eddahibi et al. 2006).

In contrast, vasodilator molecules are reduced. On the one hand, NO, a vasodilator gas, is synthesized from L‐arginine by the enzyme nitric oxide synthase (NOS). NO, which induces VSMCs relaxation and inhibits proliferation through upregulation of the cell cycle inhibitor p21 and inactivation of RhoA/ERK signaling, is reduced in idiopathic PAH (iPAH) patients. However, there is a controversy about the expression of the endothelial isoform of NOS (eNOS), as it may not reflect NO levels, since the enzyme becomes uncoupled and produces superoxide (Gallardo‐Vara et al. 2023). In this context, it is worth mentioning the importance of caveolin1, a major component of caveolae, which is known to induce chronic activation of eNOS and production of peroxynitrite, a vasoconstrictor, and has been seen to be downregulated in monocrotaline‐induced PH and PAH patients (Méndez‐Barbero et al. 2021). On the other hand, ECs are also the primary source of prostacyclin, a molecule produced through an enzymatic process involving cyclooxygenase (COX) and prostacyclin synthase, starting from arachidonic acid. Once released by ECs, prostacyclin binds to the I‐prostanoid receptor, a G protein‐coupled receptor (GPCR), located on SMCs. This binding activates adenylate cyclase, converting adenosine triphosphate (ATP) into the second messenger cyclic adenosine monophosphate (cAMP), which in turn activates protein kinase A (PKA) and promotes smooth muscle relaxation and vasodilation. In addition, prostacyclin inhibits VSMCs proliferation and has anti‐thrombotic and anti‐inflammatory actions. However, in PH, this pathway becomes dysregulated, shifting towards the production of thromboxane A2, a molecule that promotes platelet aggregation, vasoconstriction, and smooth muscle proliferation, contributing to disease progression (Luo and Qiu 2022).

#### 3.1.2 Cell‐To‐Cell Direct Contact

Direct cell‐to‐cell contact between ECs and VSMCs is also an essential component of vascular homeostasis. There is a lot of research around the Notch signaling in the ECs‐VSMCs communication. Notch is a well‐conserved signaling mechanism needed for angiogenesis. For its activation, it needs the interaction between the membrane Notch receptors (Notch 1–4) and the membrane Notch ligand (Jagged 1,2 and Delta‐like 1,3 and 4) (Méndez‐Barbero et al. 2021).

In PAH patients, it has been described an upregulation of Notch3 in lung SMCs. This Notch3 signaling pathway has been studied as a regulator of the phenotypic switch in VSMCs, promoting dedifferentiation and elevated migration and proliferation (Thistlethwaite et al. 2010; Ramadhiani et al. 2023). It has been reported that senescent ECs can interact with VSMCs via Notch‐mediated signaling contributing to the pathogenesis of PAH (Ramadhiani et al. 2023). This might be more relevant in elderly patients, in whom the incidence and prevalence of PH arise. Additionally, it has been reported an upregulation of Piezo1, a mechanosensitive cation channel, in ECs from patients with iPAH and PH animal models. Piezo1 is activated through membrane stretch, which increases the Ca^+2^ influx and activates signaling pathways, including ERK/MAPK and AKT/mTOR signaling. This event results in the upregulation of Notch ligands and, thus, may contribute to the phenotypic switch of SMCs (Wang et al. 2021).

#### 3.1.3 EC Derived Extracellular Vesicles

Extracellular vesicles (EVs), including exosomes, microvesicles and apoptotic bodies, refer to cell‐derived membranous structures that play a crucial role in intercellular communication and multiple biological processes, such as immune regulation, apoptosis and vascular remodeling. EVs have been studied as biomarkers and pathogenic effectors in PH and, particularly, those derived from ECs (EEVs) have gained significant attention. Circulating EEVs have been described as markers of endothelial injury and vascular remodeling. Specifically, PH patients have increased levels of EEVs expressing E‐selectin, VE‐cadherin and PECAM‐1, which correlates with increased severity of PH (Conti et al. 2023).

One of the key mechanisms by which EVs participate in PH pathogenesis is through the delivery of microRNAs (miRNAs), small non‐coding RNA molecules that influence post‐transcriptional gene expression. miRNAs negatively regulate gene expression through the recognition and binding to 3′‐untranslated regions of mRNAs, preventing their expression by blocking their translation or promoting their degradation (Deng et al. 2015). In this way, EVs can promote the overproliferative phenotype of VSMCs. For instance, hypoxic conditions increase levels of miR‐210‐3p in EEVs, which contributes to the excessive proliferation of VSMC. Although the expression of this miRNA has already been described in hypoxic VSMCs and in hypoxic‐induced PH, it is plausible that these EEVs may add an additional load to the VSMCs. Moreover, these authors also reported that the EVs may also deliver anti‐proliferative miRNAs, which suggests that the balance of miRNAs in the EVs could be modulated to exacerbate or inhibit proliferation (Chen et al. 2022). There is also evidence that EEVs can carry miR‐195, an extracellular messenger that can inhibit the expression of the serotonin transporter in VSMCs, therefore negatively controlling their proliferation (Gu et al. 2017). Additionally, other authors have also described that environmental factors, specifically cigarette smoke, can enhance the expression of miR‐1249 in EEVs, which facilitates the hyperproliferative and antiapoptotic phenotype of VSMCs exacerbating PH evolution (Su et al. 2022).

### 3.2 VSMCs to ECs Communication

As we have already mentioned, endothelial dysfunction in the vascular system is central in the development and onset of PH. Therefore, it is essential to define and characterize the different factors that may contribute to their pathological status. As previously discussed, communication between cells influences and participates in the PH progression; thus, it is relevant not only to study the influence of ECs in VSMCs but also in the opposite direction.

One important signaling pathway associated with the onset and development of PAH is the bone morphogenic protein receptor 2 (BMPR2), a transforming growth factor‐beta (TGF‐β) receptor, expressed in both VSMCs and ECs. BMPR2 and its downstream effectors, Smad1/5/8, are altered within pulmonary vascular smooth muscle and ECs, causing an imbalance of proproliferative and antiproliferative signals (Nabeh et al. 2024; Humbert et al. 2021; Atkinson et al. 2002). Loss‐of‐function mutations in the *BMPR2* gene are the most common drivers of hereditary PAH, and reduced expression of BMPR2 has been observed in iPAH (Humbert et al. 2022; Nabeh et al. 2024). Dysfunction of this receptor alters ECs' metabolism, which leads to apoptosis and loss of factors that regulate VSMCs' proliferation. Moreover, recent evidence has shown that VSMCs can drive endothelial regeneration through a BMPR2‐Notch1 mediated via. Specifically, these authors showed that, in SMC‐EC co‐culture, through a contact‐mediated communication, BMPR2 activates EC‐Notch 1 to induce endothelial proliferation. The mechanism involves BMPR2‐dependent production of collagen IV that leads to the activation of different proteins, including Notch1 in ECs (Miyagawa et al. 2019). In this study, the authors suggest a modulation of VSMCs over ECs' proliferation and regeneration, and, therefore, maintenance of the monolayer integrity.

VSMCs are also able to communicate through EVs with the endothelium. For instance, it has been described a potential positive feedback loop generated between these two types of cells. The authors reported that VSMCs stimulated upon PDGF signaling, which, as we mentioned before, its endothelial secretion is increased under PH pathogenesis, showed increased expression of miR‐409‐5p. This miRNA is transported to ECs in EVs, and it promotes endothelial impaired function, including reduced NO production (Heo and Kang 2024). Moreover, in blood vessels, there is one miRNA cluster, the miR‐143/145, that has a key role in VSMCs differentiation and disease. miR‐145 expression has been associated with the modulation of VSMCs phenotype, since it restores the contractile phenotype in other vascular diseases such as atherosclerosis. In PH, the miR‐145‐5p was associated with neointimal lesions and observed to be upregulated in VSMCs in lung tissue from patients with heritable and iPAH. On the other hand, miR‐143, which is upregulated under hypoxia, has been found to act as a paracrine signaling mediator, since it can be delivered from VSMCs to ECs through exosomes inducing ECs migrative phenotype (Deng et al. 2015).

### 3.3 Communication Between Fibroblasts and Other Vascular Cells

Experimental data suggests that, in response to a vascular stress, adventitial fibroblasts act as sentinel cells. They are among the first cells to be activated, responding through proliferation, upregulation of contractile and ECM proteins, and the release of factors that promote VSMCs' growth and the recruitment of inflammatory cells (Pugliese et al. 2015).

Studies have also shown that EC‐fibroblast crosstalk contributes to the pathogenesis of PH. As already mentioned, ECs can become fibroblast‐like cells through EndoMT. In addition, ECs promote fibroblast migration and proliferation through the secretion of different factors, such as ET1, PDGF, and CXCL12. Endothelial production of ET1 and IL6 also induces fibroblasts' differentiation into myofibroblasts. On the other hand, the accumulation of myofibroblasts and the consequent increased stiffness of the vessel wall enhance ECs' proliferation. Activated fibroblasts further promote endothelial proliferation by releasing the metalloproteinase 2 (MMP2) and MMP9. They also secrete thrombospondin‐1, which disrupts EC‐EC interactions promoting an injured endothelium (Evans et al. 2021).

Furthermore, it has been described that fibroblasts can modulate VSMCs cell state through the production of ECM components and soluble ligands. In this way, changes in the laminin‐to‐collagen ratio due to the activation of fibroblasts alter the migration, attachment, and expansion of VSMCs. Additionally, it has been described a downregulation of pentraxin‐3 (PTX3), a protective factor, in the secretome of activated fibroblasts. Fibroblast growth factor (FGF) regulates SMC survival, proliferation, and migration, and its endothelial production is increased during PH (Evans et al. 2021). Since PTX3 inhibits the proliferative action of this FGF and its expression, its loss reduces protection against vascular lesions (Crnkovic et al. 2025; Dai et al. 2025).

### 3.4 Communication Between Vascular and Immune Cells

PH is increasingly recognized as a disease associated with inflammation, emphasizing the relevance of cell communication between immune cells and vascular cells in its pathogenesis. Evidence from experimental models suggests that perivascular inflammation often precedes the aberrant vascular remodeling, underscoring the immune system's key contribution to the structural changes characteristic of PH (Liu et al. 2022). Moreover, the persistent accumulation of monocytes and macrophages in the perivascular area has been consistently observed across different animal models and human cases, therefore becoming a hallmark of PH. This inflammation in the adventitia has also shown a positive correlation with mean pulmonary artery pressures (Zhang, Li, et al. 2024; Stacher et al. 2012). As mentioned before, the cell state of fibroblasts is altered during the pathogenesis of PH. It has been demonstrated that, in hypoxia‐induced pulmonary vascular remodeling, there is a specific subpopulation of fibroblasts that show a constitutively activated pro‐inflammatory phenotype able to drive the recruitment and activation of monocytes and macrophages (Zhang, Li, et al. 2024).

Moreover, circulating levels of inflammatory mediators are associated with worse clinical outcome in PAH. In this way, it is demonstrated that pulmonary vascular cells are not only targets of inflammation, but also key sources of inflammatory signals, as they acquire pathological pro‐inflammatory phenotypes (Humbert et al. 2019). For instance, in PAH, ECs display increased surface expression of adhesion molecules such as E‐selectin, intercellular adhesion molecule 1 (ICAM1), and vascular cell adhesion molecule 1 (VCAM1). They also secrete elevated levels of pro‐inflammatory cytokines (e.g., IL‐1β, IL‐6, and TNFα) and chemokines. This creates a feedback loop that drives immune cell recruitment, vascular remodeling, and disease progression (Humbert et al. 2019; Liu et al. 2022; Heo and Kang 2024). In addition, inflammation promotes EndMT, which is triggered by cytokines such as IL‐1β and TNF‐α. EndMT cells further amplify inflammation by secreting higher concentrations of cytokines like IL‐4, IL‐6, IL‐8, IL‐13, and TNF‐α, perpetuating the feedback loop (Liu et al. 2022). Furthermore, the activated fibroblasts also contribute to this microenvironment rich in proinflammatory cytokines through the expression of IL‐1β and IL‐6, among others (Zhang, Li, et al. 2024; Dai et al. 2025).

Hypoxia, a hallmark feature of PH, exacerbates this inflammatory environment. Hypoxic conditions stimulate nuclear factor‐kappa B (NF‐κB), promoting the production of pro‐inflammatory cytokines. Moreover, inflammation itself can create hypoxic environments in tissues. This bidirectional relationship, mediated by HIFs, reinforces the close crosstalk and relevance of inflammation and PH progression (El Alam et al. 2022).

#### 3.4.1 Role of the Different T‐Cells Subsets

Different T cell subtypes, key components of the adaptive immune system, differentially contribute to PH pathogenesis, either exacerbating or mitigating the disease progression.

Among these subtypes, cytotoxic CD8+ T cells accumulate in peripheral lung tissue in PH. Despite reduced levels in plasma, these cells promote disease progression through their cytolytic activity triggering local inflammatory responses (Luo and Qiu 2022). Similarly, helper T cells (CD4+), particularly Th1 and Th17 subsets, contribute to PH progression through the secretion of pro‐inflammatory cytokines such as IL‐6, IL‐2, IL‐21, interferon‐gamma (IFN‐γ), and TNF‐α. Th2 cells have also been reported to exacerbate PAH by producing IL‐4 and IL‐13, which further drive vascular remodeling and inflammation (Qiu et al. 2019). In this context, it has been suggested that fibroblasts contribute to the increased inflammatory polarization and activation of CD4+ T cells (Plecitá‐Hlavatá et al. 2023).

Contrary, regulatory T‐cells (Treg) provide a protective and immunosuppressive effect, which counteracts the pro‐inflammatory activity of the other cells involved in the pathogenesis. Under physiological conditions, Tregs are in balance with other subsets, but alterations in the Treg/Th17 ratio have been seen in idiopathic PAH, hereditary PAH, and PAH associated with connective tissue diseases, correlating with disease severity. Interestingly, elevated Treg levels have been observed in some studies as a potential compensatory response to suppress peripheral inflammation (Qiu et al. 2019). Tregs mitigate PH progression through several mechanisms. First, they help preserve pulmonary EC function by secreting IL‐10, which enhances eNOS phosphorylation and reduces oxidative stress by inhibiting NADPH oxidase activity, leading to improved vascular relaxation and protection of pulmonary arterioles. Second, Tregs inhibit VSMCs proliferation by the release of anti‐inflammatory cytokines and suppressing proliferative signaling pathways such as AKT and ERK (Qiu et al. 2019). In animal models, Tregs have been shown to reduce VSMCs hyperplasia, lower right ventricular systolic pressure, and attenuate hypoxia‐induced PH. For instance, in athymic rats, reconstitution with CD4 + CD25+ Tregs significantly reduced vascular remodeling and PH progression induced by the VEGF receptor blocker SU5416, highlighting the therapeutic potential of Tregs (Tamosiuniene et al. 2011).

#### 3.4.2 Communication With Macrophages

Macrophages are innate immune cells that can dynamically interact with the vascular cells. These cells can polarize into two subsets, M1 and M2, with different functions in PH pathogenesis. These subsets are characterized by distinct surface cell markers and released cytokines. In this way, M1 macrophages have a pro‐inflammatory phenotype, while M2 are involved in anti‐inflammatory and wound healing responses (Luo and Qiu 2022).

Lung macrophages can be divided into alveolar macrophages (AMs) and interstitial macrophages (IMs). AMs derive from embryonic cells and maintain immune homeostasis, while IMs originate from circulating monocytes and participate in adaptive immune responses. In PH patients, monocyte‐derived macrophages are recruited to the lungs in response to inflammatory signals and are often the first immune cells detected in pulmonary vascular lesions (Zuo et al. 2024). These cells are mainly accumulated in perivascular areas, and the infiltrated monocytes react to microenvironmental changes in the lungs coordinating the inflammatory response (Li et al. 2021). It has been reported that, under the initial hypoxic exposure, both AMs and IMs release pro‐inflammatory cytokines. Over time, the macrophages' role shifts, with perivascular IMs promoting tissue repair and remodeling, while AMs maintain a pro‐inflammatory phenotype. Accordingly, in experimental PH models, it has been shown that, during early stages, M1 macrophages predominate promoting inflammation, whereas M2 macrophages become prevalent during later stages with increased M2/M1 ratio (Zuo et al. 2024). Moreover, ex vivo studies have shown that fibroblasts from PH patients and animal models can recruit, retain, and, activate monocytes/macrophages towards this proinflammatory and proremodelling phenotype through the liberation of cytokines (Kumar et al. 2021; Dai et al. 2025; Li et al. 2021). Beyond these soluble mediators, it has also been demonstrated that EVs released from adventitial fibroblasts are key mediators in the complement‐dependent proinflammatory activation of macrophages (Kumar et al. 2021).

M2 macrophages exacerbate the disease by stimulating VSMCs growth and proliferation via the CX3CL1/CX3CR1 axis. Additionally, the macrophages accumulating in the lungs release leukotrienes (LTs), particularly LTB4, which induces endothelial apoptosis and VSMCs proliferation (Luo and Qiu 2022). Moreover, macrophages also produce PDGF and VEGF, further amplifying the proliferation of VSMCs (Heo and Kang 2024). Lung‐resident cells produce IL‐4 and IL‐13, which polarize macrophages towards the M2 phenotype, exacerbating the vascular remodeling (Luo and Qiu 2022).

Additionally, as previously mentioned, the BMPR2 gene alteration underlies many heritable PAH cases, and it has been shown that the loss of this receptor in ECs induces GM‐CSF production in response to TNFα. Fibroblasts also secrete this macrophage activation factor GM‐CSF. GM‐CSF facilitates macrophage recruitment and promotes the secretion of pro‐inflammatory cytokines, further driving vascular dysfunction. Consistent with this, elevated GM‐CSF levels have been reported in iPAH, underscoring the importance of this pathway (Zhang, Li, et al. 2024; Luo and Qiu 2022).

Chemokine signaling systems are essential in macrophage recruitment and communication with vascular cells in PH. In this way, CCL2‐CCR2, CCL5‐CCR5, and CX3CL1‐CX3CR1 pathways are particularly important in mediating macrophage infiltration into pulmonary lesions and mediating vascular remodeling. Both macrophages and VSMCs express receptors for these chemokines, suggesting bidirectional interactions between both cell types (Abid et al. 2019). Additionally, ECs are essential in PH onset, since they produce proinflammatory molecules driven by HIF‐2α expression, such as CXCL12, CXCR4, ICAM1, and VCAM1, which recruits monocytes during the early stages of PH (Zuo et al. 2024; Bordenave et al. 2020). Fibroblasts also produce macrophage chemoattractant cytokines such as CCL2, CXCL12, and CCL5, as well as the co‐stimulatory molecule CD40L and the adhesion molecule VCAM1 (Zhang, Li, et al. 2024).

#### 3.4.3 Other Immune Cells and Derived Extracellular Vesicles

Neutrophils are early responders of the inflammation process and contribute to vascular remodeling through the release of molecules such as myeloperoxidase (MPO). MPO catalyzes the production of reactive oxygen species (ROS), activating vascular Rho‐kinase, which drives VSMC proliferation and vasoconstriction. Elevated plasma MPO levels have been associated with poor outcomes in PAH patients. Mast cells, other immune cells, contribute to PH development by the secretion of tryptase and chymase, which induce VSMCs proliferation and migration. On the other hand, dendritic cells (DC), which are professional antigen‐presenting cells, produce CX3CL1 chemokine, also promoting proliferation in VSMCs. Interestingly, antibodies produced by B‐cells against pulmonary fibroblasts and ECs have been reported in patients with PAH. Autoantibodies against ECs may stimulate production of adhesion molecules and cytokines, contributing to PH pathogenesis (Luo and Qiu 2022).

While EV‐mediated communication in PH remains to be further explored, preliminary evidence suggests that immune cell derived EVs, particularly those from T cells, are upregulated in various forms of PH. However, their specific molecular content and role in PH pathogenesis have not been characterized. Future studies should focus on identifying molecules delivered by these vesicles and their contributions to disease progression (Kosanovic et al. 2019).

## 4 The Relevance of the Pulmonary Microenvironment

Current used treatments in clinics against PH can be divided into four classes aimed at restoring the vasoactive homeostasis: (1) phosphodiesterase inhibitors (sildenafil, tadalafil, vardenafil) that target the NO signaling pathway; (2) the guanylate cyclic activator Riociguat; (3) prostacyclin analogs (treprostinil, iloprost, epoprostenol); (4) and endothelin receptor antagonists (ambrisentan, macitentan, bosentan) (Dunmore et al. 2021). Recently, new strategies targeting the inflammation side of the disease, such as B‐cell depletion with rituximab or anti‐IL‐6 therapies such as tocilizumab, have been developed and studied (Humbert et al. 2019; Kuebler et al. 2018; Zhang, Liu, et al. 2024). However, only one treatment, sotatercept, directly targets vascular remodeling. Specifically, sotatercept is a fusion protein of the activin receptor (ACTRIIA) and the Fc domain of human IgG1. Its mechanism of action implicates sequestering free activins thus modulating the Smad pathway, which is related to the already mentioned BMPR2 receptor (Humbert et al. 2021; Savale et al. 2024). Therefore, sotatercept rebalances SMAD signaling and proliferative behavior in PAH pathogenesis (Kumar et al. 2024; Humbert et al. 2021; Condon et al. 2022). Apart from being a promising therapy on its own for targeting a pathogenic pathway different from preexisting therapies, the combination of these therapies in addition to sotatercept could enhance therapeutic effectiveness in PAH (Humbert et al. 2023). Thus, exploring the dynamic communications between the immune cells and vascular cells provides a more integrative perspective to its understanding and management that may be useful designing combined therapies with disease‐modifying potential.

PH pathological features can be compared to those seen in cancer, such as excessive proliferation, resistance to apoptosis, inflammation and fibrosis. These similarities highlight the potential of borrowing and applying advances and concepts from cancer research to PH understanding (D'Alessandro et al. 2018; Zhang et al. 2023). As in cancer progression, the disease onset is influenced by the surrounding cells and their interactions, which sets a promising concept, the pulmonary microenvironment, as a novel way of understanding PH. Tumor microenvironment refers to the ecosystem generated by the complex interactions between cancer cells and stromal elements, such as immune cells and soluble molecules like chemokines and cytokines (Anderson and Simon 2020; Xiao and Yu 2021; Mayer et al. 2023). This malignant microenvironment supports cancer cell survival and invasion, playing a decisive role in the tumor progression and maintenance (Anderson and Simon 2020; Baghban et al. 2020). Therefore, if we translocate the microenvironment concept to PH disease, it would be defined as the complex network of interactions between the “malignant” vascular cells and stromal components that supports the aberrant growth and pathological phenotypes of the three vessel layers. In this way, complexity increases if we also consider the cardiac microenvironment, where emerging research has emphasized the importance of intercellular communication involving fibroblasts, immune cells and ECs (Bazgir et al. 2023). Moreover, lung‐heart communication might also be a critical factor to be considered, as right ventricle failure often drives the patient outcome. Some authors have studied the importance of this crosstalk and, recently, it has been suggested that exosomal miRNAs can facilitate lung‐heart communication, potentially participating in cardiac dysfunction (de Man and Vonk‐Noordegraaf 2021; Chang et al. 2023). However, evidence is very limited and further research is needed to describe their function.

Advanced technologies like single‐cell transcriptomics have already started describing the intercellular crosstalk in PH, showing how VSMCs and immune cells interact abnormally driving vascular remodeling (Crnkovic et al. 2022). Additionally, as previously described, the behavior of immune cells like macrophages varies over time, underscoring the need to study these interactions across different disease stages. All together highlights the importance of studying PH as a dynamic system, both within the lungs and in their communication with the heart. This integrative perspective and the application of advanced technologies can reveal new therapeutic targets to not only stop disease progression but also restore homeostasis within this complex system.

## 5 Conclusions

In conclusion, cellular crosstalk between the immune and vascular systems is central in the pathological cascade underlying vascular remodeling in PH. The consideration of the pulmonary microenvironment as a pathogenic factor itself sets the basis for the study of PH from a more integrative and wider perspective. This will not only expand our knowledge on the disease, but will also open the path for combined therapeutical strategies. Despite the recent clinical advances, further research is still needed to find an effective cure, which could benefit from this multifactorial view. Further studies should be conducted to describe this complex network and identify the key potential targets. Moreover, other components, such as ECM and pneumocytes, should also be considered as relevant participants in the pulmonary microenvironment.

## Author Contributions

Conceptualization: Lola Navarro‐Llinares and Eduardo Oliver. Writing – original draft: Lola Navarro‐Llinares. Designing and drawing figures: Lola Navarro‐Llinares and Laura de la Bastida‐Casero. Writing – review and editing: Lola Navarro‐Llinares, Bertha García‐León, Laura de la Bastida‐Casero, and Eduardo Oliver. Funding acquisition: Eduardo Oliver. Supervision: Eduardo Oliver. All authors have read and agreed to the published version of the manuscript.

## Funding

This work was supported by funding from Ministerio de Ciencia, Innovación y Universidades/Agencia Estatal de Investigación MCIN/AEI/10.13039/501100011033 and by “ERDF A way of making Europe” (PID2021‐123167OB‐I00, PID2024‐159407OB‐I00 and RED2022‐134299‐T), CSIC Talent Attraction program (20222AT010) and Ayudas especiales para la preparación de proyectos (2025AEP161), CIBERCV‐CIBERNED collaborative funds (CV24PI04/2024), La Marató de TV3 Foundation (202336‐30‐31) and Ayudas para la investigación de la Fundación contra la Hipertensión Pulmonar. L.B.‐C. was a recipient of a predoctoral fellowship from Comunidad de Madrid (PIPF‐2022/SAL‐GL‐24824). B.G.‐L. was a recipient of a Severo Ochoa FPI predoctoral fellowship from Ministerio de Ciencia e Innovación/Agencia Estatal de Investigación (PRE2022‐104403).

## Conflicts of Interest

The authors declare no conflicts of interest.

## Data Availability

The authors have nothing to report.
